# Supplementary material for: Analysis of Changes in Antibiotic Use Patterns in Korean Hospitals during the COVID-19 Pandemic
Source: Antibiotics (Basel). 2023 Jan 18;12(2):198. doi: 10.3390/antibiotics12020198 (PMC9952207; doi:10.3390/antibiotics12020198)
Supplement: Supplementary file 1 [file antibiotics-12-00198-s001.zip › Supplementary_Table_20230109.pdf]

Table S1. Classification of antimicrobial agents

**Classification**	**Antimicrobial agents**
total antibacterial agents	All
broad-spectrum antibacterial agents predominantly used for hospital-onset infections	Amikacin, Tobramycin, Cefepime, Cefpirome, Ceftazidime, Cefoperazone/sulbactam, Imipenem, Meropenem, Doripenem, Piperacillin/tazobactram, Piperacillin/sulbactam
broad-spectrum antibacterial agents predominantly used for community-acquired infections	Cefdinir, Cefixime, Cefotaxime, Cefpodoxime, Ceftriaxone, Cefcapene, Cefditoren, Cefetamet, Cefodizime, Cefpiramide, Ceftizoxime, Ertapenem, Gemifloxacin, Levofloxacin, Moxifloxacin, Ciprofloxacin, Lomefloxacin, Norfloxacin, Ofloxacin, Tosufloxacin
antibacterial agents predominantly used for resistant gram-positive bacterial infections	Linezolid, Vancomycin (parenteral), Teicoplanin, Daptomycin
narrow-spectrum beta-lactam agents	Amoxicillin, Amoxicillin and beta-lactamase inhibitor, Amoxicillin/clavulanate, Amoxicillin/sulbactam, Ampicillin, Ampicillin/sulbactam, Nafcillin, Sultamicillin, Benzylpenicillin, Cefadroxil, Cefazolin, Cefalexin, Cefotetan, Cefoxitin, Cefaclor, Cefprozil, Cefradine, Cefroxadine, Cefuroxime, Cefamandole, Cefazedone, Cefmetazole, Cefminox, Cefotiam, Ceftezole, Flomoxef
antibacterial agents predominantly used for extensive antibiotic-resistant gram-negative bacterial infections	Colistin, Tigecycline, Ceftolozane/tazobactam
carbapenem	Imipenem, Meropenem, Doripenem, Ertapenem
fluoroquinolone	Gemifloxacin, Levofloxacin, Moxifloxacin, Ciprofloxacin, Lomefloxacin, Norfloxacin, Ofloxacin, Tosufloxacin
metronidazole	Metronidazole

Table S2. Distribution of the number of COVID-19 patients in Korean hospitals.

	Number of patients
10 Percentile	20.1
20 Percentile	33.0
30 Percentile	47.3
40 Percentile	87.4
50 Percentile	132.5
60 Percentile	211.2
70 Percentile	305.0
80 Percentile	480.8
90 Percentile	954.3
100 Percentile	4,164

Table S3. *ICD-10* codes for all-cause pneumonia

ICD-10 **codes**	**Disease description**		
**J10.0, J11.0**	Influenza pneumonia		
**J12.0-2, J12.8, J12.9**	Viral pneumonia:		
	J12.0 (Adenoviral)	J12.1 (RSV)	J12.2 (parainfluenza)
	J12.8 (Other viral)	J12.9 (Viral pneumonia, unspecified)	
**J13**	Pneumonia due to *Streptococcus pneumoniae*		
**J14**	Pneumonia due to *Haemophilus influenza*		
**J15.0-9**	Bacterial pneumonia:		
	J15.0 (*Klebsiella pneumoniae*)	J15.1 (*Pseudomonas* spp.)	J15.2 (*Staphylococcus* spp.)
	J15.3 (*Streptococcus*, group B)	J15.4 (Other *Streptococci*)	J15.5 (*E. coli*)
	J15.6 (Other aerobic gram-negative)	J15.7 (*Mycoplasma pneumoniae*)	J15.8 (Other bacterial)
	J15.9 (Bacterial pneumonia, unspecified)		
**J16.0, J16.8**	Other infectious organisms		
	J16.0 (Chlamydia)	J16.8 (Other specified infectious organisms)	
**J17.0-3, J17.8**	Pneumonia in diseases classified elsewhere:		
	J17.0 (Pneumonia in diseases)	J17.1 (Pneumonia in viral diseases)	
	J17.2 (Pneumonia in mycoses)	J17.3 (Pneumonia in parasitic diseases)	
	J17.8 (Pneumonia in other diseases)		
**J18.0-2, J18.8, J18.9**	Pneumonia, organism unspecified:		
	J18.0 (Bronchopneumonia)	J18.1 (Lobar pneumonia)	
	J18.2 (Hypostatic pneumonia)	J18.8 (Other pneumonia)	J18.9 (Pneumonia)
**A481**	Legionella pneumonia		

Table S4. COVID-19 patient management hospitals, the total number of COVID-19 patients in the bottom 10% hospital.

	Pre-COVID	Post-COVID	Change in level	SE	95% CI	*P*	Change in trend	SE	95% CI	*P*
Total antibacterial agents	678.42	641.29	23.79	24.28	-26.85 to 74.44	0.339	-8.65	3.11	-15.14 to -2.16	0.012
Broad-spectrum antibiotics predominantly used for hospital-onset infections	102.26	111.78	21.67	4.71	11.84 to 31.50	<0.001	0.10	0.71	-1.39 to 1.59	0.890
Broad-spectrum antibiotics predominantly used for community-acquired infections	292.57	274.69	0.75	13.41	-27.22 to 28.72	0.956	-2.46	1.58	-5.75 to 0.83	0.135
Antibacterial agents predominantly used for resistant gram-positive bacterial infections	22.06	26.11	10.85	2.02	6.62 to 15.07	<0.001	0.46	0.32	-0.22 to 1.14	0.173
Narrow-spectrum beta-lactam agents	117.74	109.76	5.10	3.23	-1.63 to 11.83	0.130	-2.67	0.38	-3.49 to -1.89	<0.001
Antibacterial agents predominantly used for extensive antibiotic-resistant gram-negative bacterial infections	2.36	2.52	2.17	0.38	1.38 to 2.96	<0.001	-0.04	0.05	-0.15 to 0.07	0.432
Carbapenem	22.23	27.48	9.13	2.36	4.21 to 14.04	0.001	-0.24	0.33	-0.92 to 0.45	0.475
Fluoroquinolone	93.05	83.61	9.18	6.43	-4.23 to 22.59	0.169	-0.17	0.87	-1.98 to 1.64	0.846
Metronidazole	39.89	39.28	0.19	2.36	-4.74 to 5.11	0.938	-0.17	0.35	-0.90 to 0.56	0.633

Abbreviations: COVID, Coronavirus disease 2019; SE, Standard error; CI, Confidence interval

Table S5. COVID-19 patient management hospitals, the total number of COVID-19 patients in the top 10% hospitals.

	Pre-COVID	Post-COVID	Change in level	SE	95% CI	*P*	Change in trend	SE	95% CI	*P*
Total antibacterial agents	656.05	571.93	33.81	36.09	-41.48 to 109.10	0.360	-15.89	5.40	-27.15 to -4.63	0.008
Broad-spectrum antibiotics predominantly used for hospital-onset infections	101.79	96.73	14.98	7.00	0.37 to 29.58	0.045	-1.67	1.12	-4.00 to 0.65	0.149
Broad-spectrum antibiotics predominantly used for community-acquired infections	277.03	252.12	15.85	12.83	-10.91 to 42.62	0.231	-5.14	1.83	-8.95 to -1.34	0.011
Antibacterial agents predominantly used for resistant gram-positive bacterial infections	29.62	30.04	12.12	3.64	4.54 to 19.71	0.003	-0.33	0.62	-1.63 to 0.97	0.607
Narrow-spectrum beta-lactam agents	106.93	88.88	-4.08	9.70	-24.31 to 16.16	0.679	-4.64	1.28	-7.30 to -1.98	0.002
Antibacterial agents predominantly used for extensive antibiotic-resistant gram-negative bacterial infections	4.61	3.98	1.93	0.64	0.59 to 3.26	0.007	0.19	0.13	-0.09 to 0.47	0.165
Carbapenem	35.18	33.29	7.34	2.56	2.01 to 12.67	0.009	-0.46	0.51	-1.52 to 0.59	0.372
Fluoroquinolone	102.14	98.60	11.94	8.96	-6.75 to 30.63	0.198	-0.10	1.03	-2.25 to 2.04	0.922
Metronidazole	37.21	27.86	0.53	3.80	-7.39 to 8.45	0.890	-0.63	0.51	-1.68 to 0.43	0.228

Abbreviations: COVID, Coronavirus disease 2019; SE, Standard error; CI, Confidence interval
