# Supplementary material for: Analysis of Changes in Antibiotic Use Patterns in Korean Hospitals during the COVID-19 Pandemic
Source: Antibiotics (Basel). 2023 Jan 18;12(2):198. doi: 10.3390/antibiotics12020198 (PMC9952207; doi:10.3390/antibiotics12020198)
Supplement: Supplementary file 1 [file antibiotics-12-00198-s001.zip › Supplement_20230117.pptx]

## Slide 1
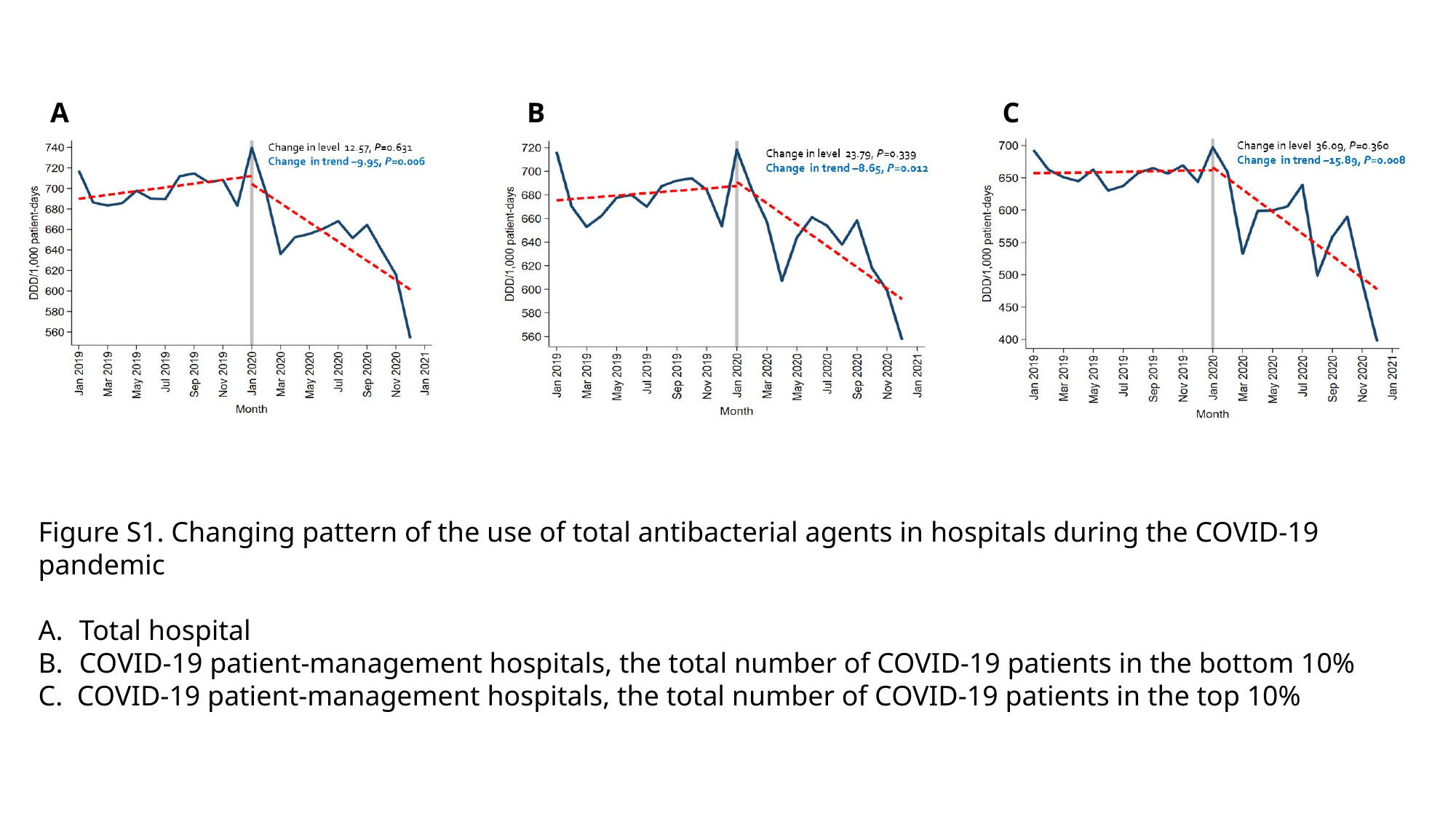

A
B
C
Figure S1. Changing pattern of the use of total antibacterial agents in hospitals during the COVID-19 pandemic
Total hospital
COVID-19 patient-management hospitals, the total number of COVID-19 patients in the bottom 10%
C. COVID-19 patient-management hospitals, the total number of COVID-19 patients in the top 10%

## Slide 2
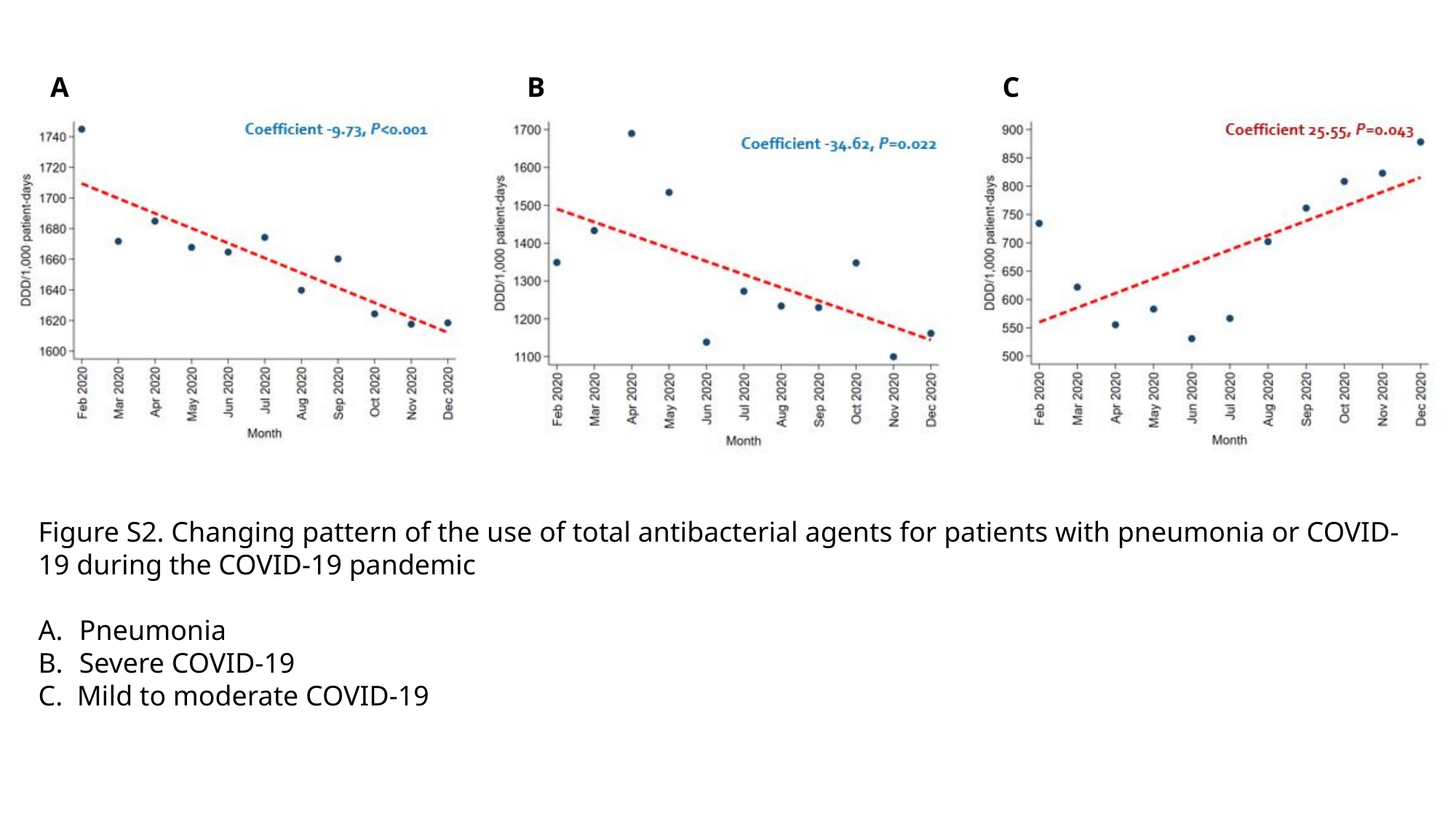

A
B
C
Figure S2. Changing pattern of the use of total antibacterial agents for patients with pneumonia or COVID-19 during the COVID-19 pandemic
Pneumonia
Severe COVID-19
C. Mild to moderate COVID-19
